# Effectiveness of Cognitive and Behavioral Interventions in the Treatment of Schizophrenia: An Umbrella Review of Meta-Analyses

**DOI:** 10.3390/jcm15010187

**Published:** 2025-12-26

**Authors:** Gabriel X. D. Tan, Andree Hartanto, Zoey K. Y. Eun, Meilan Hu, Kean J. Hsu, Nadyanna M. Majeed

**Affiliations:** 1 School of Social Sciences, Singapore Management University, Singapore 179873, Singapore; 2 Faculty of Arts and Social Sciences, National University of Singapore, Singapore 117570, Singapore

**Keywords:** intervention, cognitive, behavioral, psychotherapy, effectiveness, treatment, schizophrenia, systematic review

## Abstract

**Background:** Cognitive and behavioral interventions have risen in popularity both as an adjunctive treatment to antipsychotic medication and as an alternative treatment for schizophrenia. With the growing number of such interventions, we performed an umbrella review to provide a comprehensive summary comparing the effectiveness of the different interventions among populations with schizophrenia. **Methods:** This umbrella review included meta-analyses evaluating cognitive and behavioral interventions for schizophrenia. Following PRISMA guidelines, the initial search yielded 4888 records, and after a three-stage screening procedure, 33 meta-analyses met the inclusion criteria for the final analysis. **Results:** Our findings from the 33 meta-analyses support the efficacy of cognitive and behavioral interventions in reducing total symptoms (*Median g* = −0.38; Range *g* = −1.56 to −0.08), positive symptoms (*Median g* = −0.30; Range *g* = −0.84 to 0.00), and negative symptoms (*Median g* = −0.39; Range *g* = −0.66 to −0.09) of schizophrenia. Cognitive Behavioral Therapy, being the most common intervention studied, exhibited small to medium effects on total and positive symptom alleviation. In addition, there is evidence supporting the effectiveness of family psychoeducation combined with patient behavioral and skills training, exercise therapy, horticultural therapy, and music therapy. **Conclusions:** While our umbrella review solidifies the current evidence supporting cognitive and behavioral interventions as effective treatments for schizophrenia, it also reveals that treatment efficacy is highly dependent on the type of intervention used.

## 1. Introduction

Schizophrenia is a serious mental illness that is characterized by impairments in cognition and the presence of psychotic symptoms such as auditory hallucinations, paranoid delusional thinking, diminished emotional expression, and disorganized behavior [1,2,3]. Although schizophrenia, with a worldwide prevalence of 0.32%, is not as common as many other mental disorders, its personal and societal impacts are substantial [4]. The common effects of schizophrenia engender a decrease in the affected person’s quality of life [5], particularly in the domain of interpersonal and social relationships [6], as well as the lives of those they are surrounded by, such as family members and caregivers [7]. Moreover, there are also considerable economic costs associated with providing treatment, hospitalization, and rehabilitation for those diagnosed with schizophrenia [8,9].

When it comes to treatment, antipsychotic medication is still the most commonly used treatment method in the management of schizophrenia and its associated symptoms over the past 30 years [10,11,12,13]. These symptoms typically take the form of positive symptoms (e.g., hallucinations, delusions) [14] and/or negative symptoms (e.g., avolition, alogia) [15]. Although several studies have found antipsychotic medication to be beneficial in reducing symptom severity [16,17,18], there are still several critical issues concerning the use of antipsychotic medication as a treatment. Some common issues comprise the presence of substantial, sometimes even intolerable, side effects on patients [17,19,20], poor medication adherence [21,22,23], and poor patient responses to antipsychotic medication, leading to the eventual development of resistance to these medications, in a phenomenon known as treatment-resistant schizophrenia [24,25,26,27]. Considering the limitations of pharmacological treatment, psychological treatments—consisting of cognitive and behavioral interventions (as defined in methods below)—have seen a rise in popularity both as adjunctive treatments to antipsychotic medication and as standalone treatments [28,29].

Various forms of psychological treatments have since been explored to examine their effectiveness in managing schizophrenia, with common forms of cognitive and behavioral approaches including Cognitive Behavioral Therapy (CBT) [30,31], metacognitive training [32,33], and psychoeducation [34,35], among others. For example, meta-analyses by Bighelli et al. [36] and Jauhar et al. [30] found CBT to be beneficial in reducing total symptoms (i.e., both positive and negative symptoms) experienced by patients with schizophrenia, while a meta-analysis by Orfanos et al. [37] found group-based cognitive and behavioral interventions (consisting of CBT, metacognitive training, and psychoeducation, among others) to be effective in reducing negative symptoms specifically. In addition, studies on relatively less common cognitive and behavioral interventions—such as horticultural therapy [38] and music therapy [39]—have also found such therapies to be beneficial in alleviating total symptom outcomes.

However, despite substantial research on individual psychological interventions, the evidence base remains highly fragmented. As existing meta-analyses focus on specific intervention types, symptom domains, and incomparable effect size metrics, clinicians and researchers lack an overarching synthesis to guide practice. This fragmentation prevents a clear understanding of how cognitive and behavioral interventions perform as a broader class of treatments. Accordingly, a higher-level overview that consolidates these meta-analytic findings is needed to clarify the relative effectiveness of different cognitive and behavioral approaches for schizophrenia. To the best of our knowledge, no existing umbrella review has synthesized this diverse evidence in a unified analytic framework. The present umbrella review aims to bridge this gap by providing the first comprehensive summary that standardizes and compares the effectiveness of cognitive and behavioral interventions across schizophrenia symptom dimensions.

## 2. Materials and Methods

### 2.1. Transparency and Openness

This systematic review of meta-analyses was preregistered on PROSPERO (ID: CRD42023428163, date: 22 May 2023), and the Preferred Reporting Items for Systematic Reviews and Meta-Analyses (PRISMA) guidelines were followed for the reporting of results [40] (the PRISMA checklist can be found in the Supplementary Materials). Additional materials (e.g., supporting information and tables) used in the current umbrella review are provided in the journal’s Supplementary Materials and are also publicly available on Open Science Framework (OSF, Center for Open Science, Charlottesville, VA, USA; https://osf.io/r2ja7/, accessed on 13 December 2025). During the search and selection process, Zotero version 6.0.26 (Corporation for Digital Scholarship, Vienna, VA, USA) [41] was used to convert nbib files from PubMed into ris files, and Mendeley version 2.82.0 (Elsevier, Amsterdam, The Netherlands) [42] was used for consolidation and deduplication of records. R version 4.2.1 (R Foundation for Statistical Computing, Vienna, Austria) [43] was used to calculate effect sizes when raw means and standard deviations were involved using the package *metafor* version 4.2-0 (R Foundation for Statistical Computing, Vienna, Austria) [44], and to generate all forest plots using the package *ggplot2* version 3.4.1 (RStudio, Boston, MA, USA) [45].

### 2.2. Search Strategy

A systematic literature search was performed on five databases (*PubMed*; *EBSCOhost ERIC*; *EBSCOhost PsycINFO*; *Scopus*; *Web of Science*) using the keywords (schizo* OR “delusional disorder*” OR “brief psychotic disorder*”) AND (“*psychological treatment*” OR psychotherap* OR therap* OR “*psychological intervention*”) AND (“meta-analy*” OR “meta analy*”) for records available up to 23 May 2023, with an updated search conducted on 19 October 2024. In addition, manual searches were conducted in *Google Scholar* and *ProQuest Dissertations & Theses Global* using the keywords (*psychotherapy* AND *schizophrenia* AND *meta*) to capture additional published and unpublished literature; the search was done for the first 200 records from each source, sorted by relevance. No additional filters or date restrictions were applied to any of the searches.

### 2.3. Selection Criteria

The selection of records for inclusion in the current umbrella review was determined through three stages: (1) title and abstract screening, (2) full-text screening, and (3) quality assessment. Each stage was completed by at least two reviewers independently.

#### 2.3.1. Title and Abstract Screening

GXDT and a trained research assistant performed the screening for the initial search while GXDT and ZKYE performed the screening for the updated search. For both searches, the individuals involved independently screened for eligible records based on their title and abstracts, which were evaluated based on a preliminary set of criteria. These criteria looked at whether records (1) were available in English; (2) mentioned schizophrenia or related disorders, such as brief psychotic disorder, delusional disorder, schizoaffective disorder, schizophreniform disorder, and schizotypal personality disorder; (3) mentioned cognitive and behavioral interventions (see full-text screening below for more detail about the type of interventions considered); and (4) were meta-analyses. After duplicate records were removed, inter-rater agreement rates for each criterion were calculated. Overall agreement rate for title and abstract screening was excellent (*M* = 91%; Range = 87–99%; for breakdown by criteria see Supplementary Table S1). All disagreements were then resolved through discussion between the individuals involved at both the initial and updated search, and upon consensus irrelevant records were removed.

#### 2.3.2. Full-Text Screening

The remaining records from the initial search were then assessed by GXDT and a trained research assistant while the remaining records from the updated search were assessed by GXDT and ZKYE. The individuals involved independently screened the records based on their full texts, with disagreements being resolved through discussion. Overall agreement rate for full-text screening was good (*M* = 84%; Range = 74–98%; for breakdown by criteria see Supplementary Table S1). The selection criteria that guided the inclusion and exclusion of meta-analyses for the full-text screening were as follows:

1. Records were included if they were available in English.
2. Records were included if they were meta-analyses.
3. Meta-analyses were included if at least five primary studies were included in the analysis.
4. Meta-analyses were included if the majority (i.e., more than 50%) of their constituent samples comprised individuals with a diagnosis of schizophrenia and/or its associated disorders, according to the fifth edition of the Diagnostic and Statistical Manual of Mental Disorders (DSM-V) [46]. These consist of:
  - Schizophrenia
  - Brief psychotic disorder
  - Delusional disorder
  - Schizoaffective disorder
  - Schizophreniform disorder
  - Schizotypal personality disorder
5. Meta-analyses were included if they assessed the effectiveness of cognitive and behavioral interventions as a treatment. For the current umbrella review, we adopted a broad scope for selecting interventions to be included. We considered cognitive and behavioral interventions as “the informed and intentional application of clinical methods and interpersonal stances derived from established psychological principles for the purpose of assisting people to modify their behaviors, cognitions, emotions, and/or other personal characteristics in directions that the participants deem desirable” [46].
  - We used the term “cognitive and behavioral interventions” as an overarching label for psychological treatments grounded in cognitive, behavioral, or cognitive–behavioral principles, consistent with the definition stated above. Accordingly, we included interventions classified as cognitive, behavioral, or cognitive–behavioral in nature, provided they involved structured therapeutic procedures intended to modify thoughts, behaviors, emotions, or functional engagement. These include but are not limited to CBT [31], music therapy [39], and group-based interventions [37]. As we wanted to examine the effectiveness of such interventions on the individual specifically, interventions that do not target the patient directly (e.g., family psychoeducation without the involvement of the individual) were excluded.
  - For meta-analyses that contained a mix of both valid and invalid interventions, they were still included if information about the relevant cognitive and behavioral interventions were reported. In other words, a meta-analysis was included as long as it provided information about the efficacy of cognitive and behavioral interventions as a treatment; a meta-analysis was not included if they did not provide information about valid cognitive and behavioral interventions on its own.
6. Meta-analyses were included if they reported the effect of cognitive and behavioral interventions on symptom-related outcomes, specifically total symptoms, positive symptoms, and/or negative symptoms.
7. Meta-analyses were included if cognitive and behavioral interventions were compared to a control condition that was either a passive treatment control (e.g., wait-list control groups, treatment as usual, groups that did not receive any treatment) or active treatment control (e.g., medication, placebo). To increase the comparability of treatment effect sizes between the meta-analyses, control conditions where other forms of cognitive and behavioral interventions were used as a comparison group were not included.

No exclusions were applied due to locational or otherwise geospatial restrictions. That is, included meta-analyses could assess the effectiveness of cognitive and behavioral interventions as a treatment for schizophrenia globally, in a specific region, or in a specific country. In addition, meta-analyses were included regardless of peer review status. That is, both peer-reviewed and non-peer-reviewed meta-analyses were included.

#### 2.3.3. Quality Assessment

Meta-analyses were included if they did not contain any significant methodological bias, which was determined through quality assessment. A minimum methodological quality threshold was applied to ensure that included meta-analyses provided reliable and interpretable evidence. Accordingly, meta-analyses rated as low quality were excluded to prevent substantial bias from influencing the overall conclusions of the umbrella review [47,48,49]. Based on consensus between the authors, it was decided that only reviews with at least five “yes” responses, based on the Joanna Briggs Institute (JBI) critical appraisal instrument for Systematic Reviews and Research Syntheses [50], were included.

The quality of each meta-analysis was independently assessed by GXDT and MH using the Joanna Briggs Institute (JBI) critical appraisal instrument for Systematic Reviews and Research Syntheses [50]. The records were evaluated according to an 11-item checklist, where each item was rated according to four categories (*yes*, *no*, *unclear*, and *not applicable*) based on how closely the items adhered to each criterion. The criteria guiding methodological evaluation of each record were (1) clarity of review question, (2) use of appropriate inclusion criteria, (3) use of appropriate search strategies, (4) adequacy of sources and resources to search for studies, (5) use of appropriate criteria for appraisal of studies, (6) independent critical appraisal of studies, (7) employment of methods to minimize errors in data extraction, (8) use of appropriate data synthesis methods, (9) assessment of the likelihood of publication bias, (10) have recommendations for policy and/or practice backed by data reported, and (11) use of appropriate specific directives for new research. Each review was then given a score based on how many “yes” responses were accorded (i.e., the number of “yes” ratings out of 11). Overall agreement rate was excellent (*M* = 93%; Range = 84–100%; for breakdown by item see Supplementary Table S1), and any remaining discrepancies or disagreements were resolved through discussion between the first and second authors, with the involvement of the third author for further discussion when necessary.

### 2.4. Data Extraction

GXDT and ZKYE independently extracted information from the meta-analyses, which were: countries or regions covered by the meta-analysis, participant demographics (e.g., type of diagnosis of sample population), total number of studies, total unique sample size, type of cognitive and behavioral intervention, effect size of treatment (if the effect size reported was Cohen’s *d* or Hedges’ *g*), pooled means and standard deviations of the experimental and control groups (if the effect size reported was not Cohen’s *d* or Hedges’ *g*), heterogeneity statistic (*I*^2^) values, and the results of any subgroup analyses (if performed by the original meta-analysis). Overall agreement rate was good (*M* = 90%; Range = 82–100%; for breakdown by each extracted item see Supplementary Table S1), and disagreements were resolved through discussion between the authors. Regional classification of the different countries covered by each meta-analysis followed the country groupings by Wikimedia, Meta-Wiki [51] (see Supplementary Materials for detailed classification).

### 2.5. Conversion of Effect Sizes

For effect size analysis, we chose the standardized mean difference Hedges’ *g* as the effect size of interest [52]. Standardized—rather than raw—mean differences were chosen due to the use of different assessment scales in each meta-analysis, which required standardization for comparison. Thereafter, Hedges’ *g* was chosen over Cohen’s *d* as it corrects for bias in small sample sizes [53], which were more common among studies that focused on clinical populations due to the inherent nature of the sample. In this umbrella review, negative Hedges’ *g* values indicated improvement via symptom reduction (i.e., effectiveness favoring intervention over control) while positive Hedges’ *g* values indicated worsening of symptoms. In accordance with Cohen [54], effect sizes were categorized into little to no effect (|*g*| < 0.20), small (0.20 ≤ |*g*| < 0.50), medium (0.50 ≤ |*g*| < 0.80) and large (|*g*| ≥ 0.80).

For meta-analyses that reported effect sizes in Cohen’s *d*, we manually converted *d* into Hedges’ *g* using the formula $g=(1\;-\;\frac{3}{4(n_{1}+n_{2})-9})d$ provided at http://dlinares.org/cohend.html (accessed on 13 December 2025). In the event where a meta-analysis reported another effect size that could not be used or converted for comparison (e.g., raw mean differences), we used the means and standard deviations reported by the meta-analysis to calculate the effect size in terms of Hedges’ *g* using the *metafor* package in R. If the means and standard deviations of interest were not reported, at least two attempts were made to contact the original authors for the relevant information; if a response was not received, the meta-analysis was excluded as it could not be used.

### 2.6. Analytic Plan

Findings from the meta-analyses were synthesized narratively through investigating the efficacy of cognitive and behavioral interventions in providing beneficial effects towards schizophrenia symptom dimensions, more specifically in the dimensions of total symptoms, positive symptoms, and negative symptoms. No specific subgroup syntheses were made a priori, but we synthesized, exploratorily, the different subgroups covered by each meta-analysis (if any). We also took measures to reduce heterogeneity and therefore increase the comparability of treatment effects between the meta-analyses by: (1) specifying the type of control groups to be included (i.e., excluding studies that used other interventions as control comparisons) at the screening stage, (2) converting and standardizing the effect sizes across all the included meta-analyses after data extraction, and (3) grouping effects according to the symptom dimension outcomes used (e.g., total symptoms, positive symptoms, and/or negative symptoms).

## 3. Results

### 3.1. Search Outcome and Eligibility

**Figure 1:**
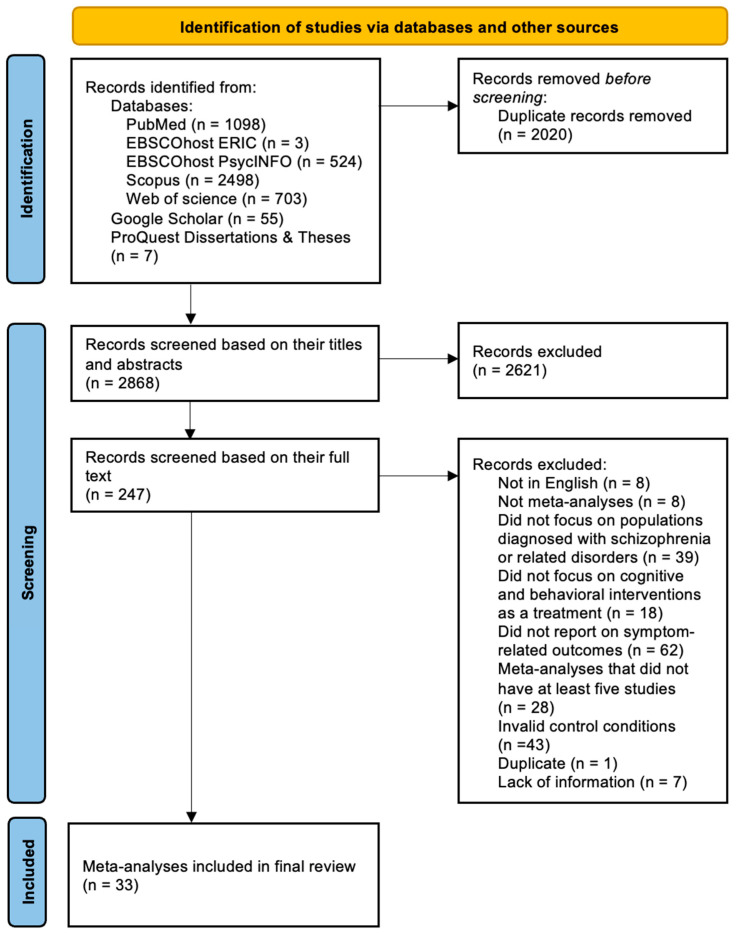
PRISMA diagram detailing all steps involved in the retrieval process with reasons for exclusion.

The search yielded a total of 2868 unique records, of which 40 met the inclusion criteria after title and abstract screening and full-text review (refer to Figure 1 for the results of the search outcome, record eligibility and evaluation). These remaining records were then assessed for their quality in order to be included in the final review.

**Figure 2:**
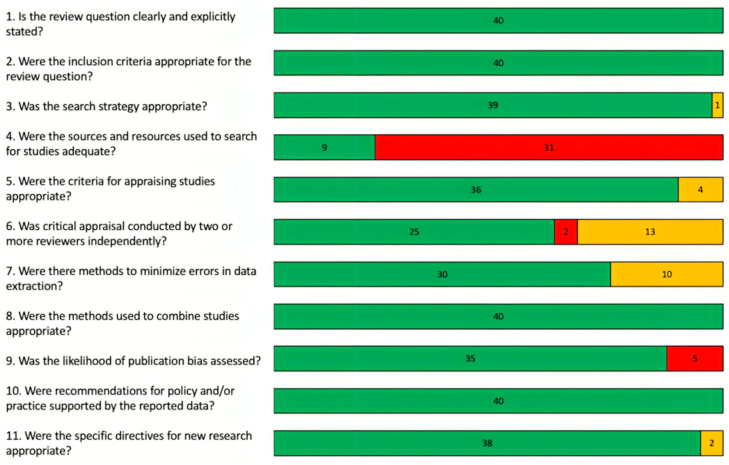
Quality assessment chart. Possible responses for each question: yes/no/unclear; Green = Yes; Red = No; Yellow = Unclear; Numbers within each bar represent the number of meta-analyses having obtained that response.

Based on the JBI critical appraisal instrument for Systematic Reviews and Research Syntheses, methodological quality scores for each meta-analysis ranged from 7 to 11 (*Median* = 9). Item four (“Were the sources and resources used to search for studies adequate?”) was found to have the lowest number of “Yes” ratings, with only nine out of the 40 meta-analyses meeting this criterion (as shown in Figure 2). This highlights the need for methodological improvement in capturing potential gray literature during searches. As all 40 meta-analyses had at least five “yes” responses, none of them had any significant methodological bias and thus met the criteria for the final inclusion.

Despite meeting the inclusion criteria and passing quality assessment, seven meta-analyses were further removed due to a lack of information (see Supplementary Materials for reasons for exclusion). This left us with an eventual 33 meta-analyses for the final review.

### 3.2. Review Characteristics and Outcomes

This umbrella review comprised 33 meta-analyses published between 2005 and 2024, with the number of primary studies included in each meta-analysis ranging from 5 to 28 (*Median* = 9) studies, indicating considerable variability in evidence base size across reviews. The included meta-analyses encompassed a broad range of psychological intervention modalities. These included cognitive–behavioral therapies (e.g., CBT and metacognitive training), activity-based or structured behavioral programs (e.g., exercise therapy, yoga therapy, and horticultural therapy), psychoeducational or skills-focused approaches (e.g., family psychoeducation with behavioral and skills training and social skills training), and other structured psychological interventions (e.g., mindfulness-based interventions, music therapy, and art therapy). These examples reflect some main intervention types but do not represent the full list of modalities examined across the included meta-analyses. The results below summarize the effectiveness of these intervention modalities across total, positive, and negative symptom domains.

#### 3.2.1. Total Symptoms

**Figure 3:**
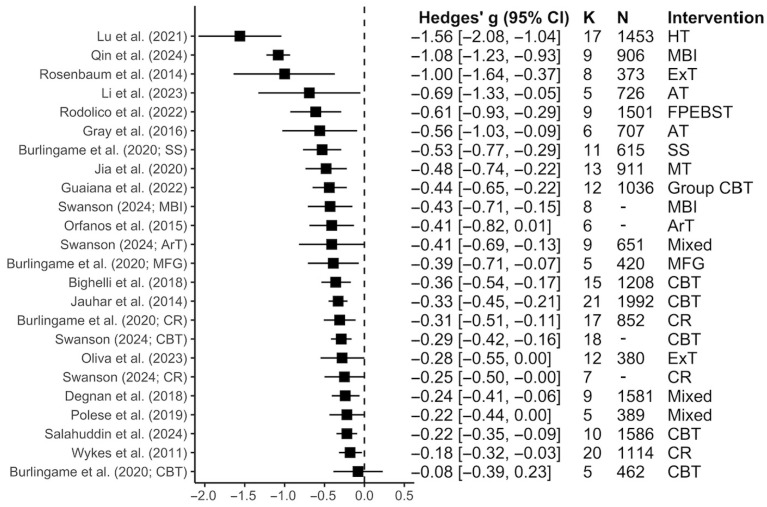
Forest plot for the effect sizes of interventions for each meta-analysis for total symptoms. *K* = number of studies; *N* = total sample size. The squares represent the point estimate of the effect size (Hedges’ *g*) for each meta-analysis, while the lines indicate their 95% CI. Additionally, the sizes of the squares are not representative of sample size. HT: Horticultural therapy; ExT: Exercise therapy; FPEBST: Family psychoeducation with patient behavioral and skills training; AT: Adherence therapy; SS: Social skills training; MT: Music therapy; CBT: Cognitive behavioral therapy; MFG: Multifamily group therapy; CR: Cognitive remediation; MBI: Mindfulness-based interventions. Meta-analyses included in this figure are: Lu et al., 2021 [38], Qin et al., 2024 [55], Rosenbaum et al., 2014 [56], Li et al., 2023 [57], Rodolico et al., 2022 [58], Gray et al., 2016 [59], Burlingame et al., 2020 [60], Jia et al., 2020 [39], Guaiana et al., 2022 [61], Swanson, 2024 [62], Bighelli et al., 2018 [36], Jauhar et al., 2014 [63], Oliva et al., 2023 [64], Degnan et al., 2018 [65], Polese et al., 2019 [28], Salahuddin et al., 2024 [66], Wykes et al., 2011 [67].

**Figure 4:**
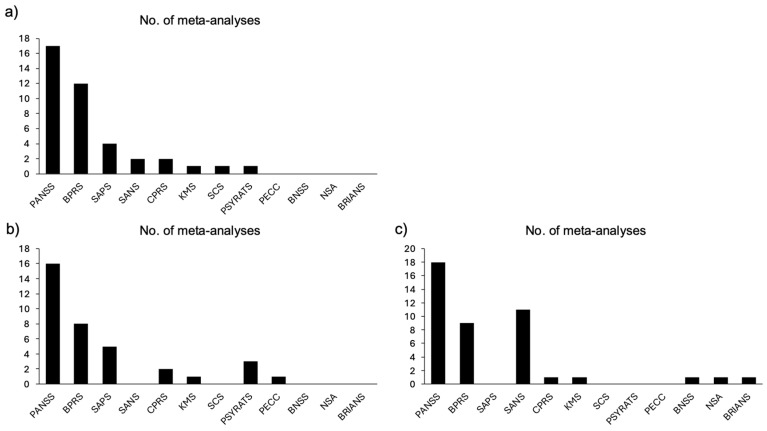
Total assessment tools used by the meta-analyses. (**a**) Total assessment tools used by the meta-analyses for total symptoms; (**b**) Total assessment tools used by the meta-analyses for positive symptoms; (**c**) Total assessment tools used by the meta-analyses for negative symptoms; PANSS: Positive and Negative Syndrome Scale; BPRS: Brief Psychiatric Rating Scale; SAPS: Scale for the assessment of positive symptoms; SANS: Scale for the assessment of negative symptoms; CPRS: Comprehensive psychopathology rating scale; KMS: Krawiecka (Manchester) scale; SCS: Strauss-Carpenter Scale; PSYRATS: Psychotic symptom rating scale; PECC: Psychosis evaluation tool for common use by caregivers; BNSS: Brief negative symptom scale; NSA: Negative symptom assessment; BRIANS: Brief assessment of negative symptoms scale.

Across the 18 meta-analyses evaluating total symptom reduction [28,36,37,38,39,55,56,57,58,59,60,61,62,63,64,65,66,67], cognitive and behavioral interventions had a small to medium effect (*Median g* = −0.38; Range *g* = −1.56 to −0.08; as shown in Figure 3). Detailed characteristics of these meta-analyses and their assessment tools can be found in Supplementary Table S2 and Figure 4a, respectively.

CBT (*k* = 6), the most frequently examined intervention, produced small to medium reductions in total symptoms (*Median g* = −0.31; Range *g* = −0.44 to −0.08). For the remaining interventions, horticultural therapy (*g* = −1.56; 95% CI = [−2.08, −1.04]), mindfulness-based interventions (*Median* g = −0.76; Range g = −1.08 to −0.43), exercise therapy (*Median* g = −0.64; Range *g* = −1.00, −0.28), and family psychoeducation with patient behavioral and skills training (*g* = −0.61; 95% CI = [−0.93, −0.29]) had the largest effect sizes (as shown in Figure 3).

#### 3.2.2. Positive Symptoms

**Figure 5:**
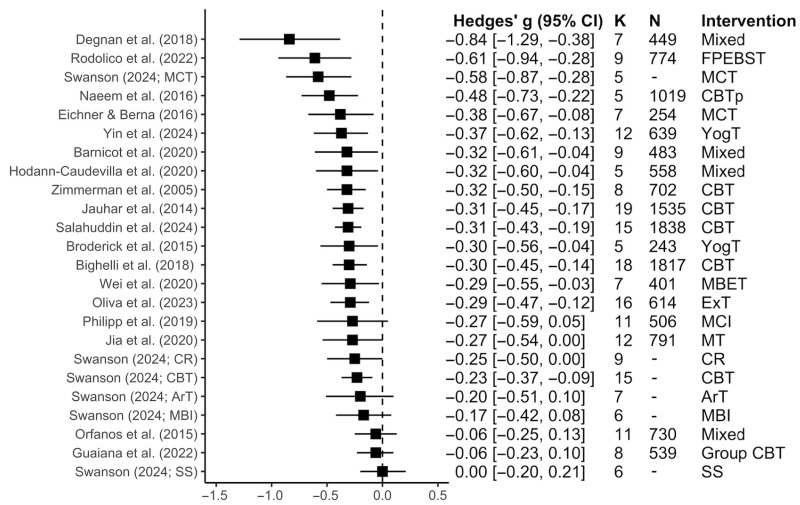
Forest plot for the effect sizes of interventions for each meta-analysis for positive symptoms. *K* = number of studies; *N* = total sample size. The squares represent the point estimate of the effect size (Hedges’ *g*) for each meta-analysis, while the lines indicate their 95% CI. Additionally, the sizes of the squares are not representative of sample size. FPEBST: Family psychoeducation with patient behavioral and skills training; MCT: Metacognitive training; CBT: Cognitive behavioral therapy; MBET: Mind–body exercise therapy; MCI: Metacognitive interventions; MT: Music therapy; YogT: Yoga Therapy; ExT: Exercise therapy; MBI: Mindfulness-based interventions. Meta-analyses included in this figure are: Degnan et al., 2018 [65], Rodolico et al., 2022 [58], Swanson, 2024 [62], Naeem et al., 2016 [68], Eichner & Berna, 2016 [69], Yin et al., 2024 [70], Bar-nicot et al., 2020 [71], Hodann-Caudevilla et al., 2020 [72], Zimmerman et al., 2005 [73], Jauhar et al., 2014 [63], Salahuddin et al., 2024 [66], Broderick et al., 2015 [74], Bighelli et al., 2018 [36], Wei et al., 2020 [76], Oliva et al., 2023 [64], Philipp et al., 2019 [75], Jia et al., 2020 [39], Orfanos et al., 2015 [37], Guaiana et al., 2022 [61].

Across the 19 meta-analyses examining positive symptom reduction [36,37,39,58,61,62,63,64,65,66,68,69,70,71,72,73,74,75,76], cognitive and behavioral interventions exhibited a small to medium effect (*Median g* = −0.30; Range *g* = −0.84 to 0.00; as shown in Figure 5). Detailed characteristics of these meta-analyses and their assessment tools can be found in Supplementary Table S3 and Figure 4b, respectively.

CBT (*k* = 7), the most frequently examined intervention, produced small to medium reductions in positive symptoms (*Median g* = −0.31; Range *g* = −0.48 to 0.00). For the remaining interventions, family psychoeducation with patient behavioral and skills training (*g* = −0.61; 95% CI = [−0.94, −0.28]) and metacognitive training (*Median g* = −0.48; Range *g* = −0.58 to −0.38) had the largest effect sizes (as shown in Figure 5).

#### 3.2.3. Negative Symptoms

**Figure 6:**
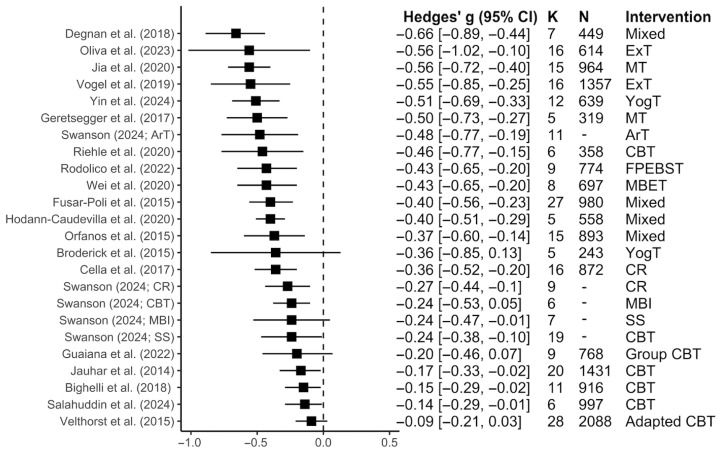
Forest plot for the effect sizes of interventions for each meta-analysis for negative symptoms. *K* = number of studies; *N* = total sample size. The squares represent the point estimate of the effect size (Hedges’ *g*) for each meta-analysis, while the lines indicate their 95% CI. Additionally, the sizes of the squares are not representative of sample size. MT: Music therapy; ExT: Exercise therapy; CBT: Cognitive behavioral therapy; FPEBST: Family psychoeducation with patient behavioral and skills training; MBET: Mind–body exercise therapy; CR: Cognitive remediation; YogT: Yoga Therapy; ExT: Exercise therapy; MT: Music therapy; ArT: Art therapy; MBI: Mindfulness-based interventions; SS: Social skills training. Meta-analyses included in this figure are: Degnan et al., 2018 [65], Oliva et al., 2023 [64], Jia et al., 2020 [39], Vogel et al., 2019 [77], Yin et al., 2024 [70], Geretsegger et al., 2017 [78], Swanson, 2024 [62], Riehle et al., 2020 [79], Rodolico et al., 2022 [58], Wei et al., 2020 [76], Fusar-Poli et al., 2015 [80], Hodann-Caudevilla et al., 2020 [72], Orfanos et al., 2015 [37], Broderick et al., 2015 [74], Cella et al., 2017 [81], Guaiana et al., 2022 [61], Jauhar et al., 2014 [63], Bighelli et al., 2018 [36], Salahuddin et al., 2024 [66], Velthorst et al., 2015 [82].

Across the 20 meta-analyses examining negative symptom reduction [36,37,39,58,61,62,63,64,65,66,70,72,74,76,77,78,79,80,81,82], cognitive and behavioral interventions exhibited a small to medium effect (*Median g* = −0.39; Range *g* = −0.66 to −0.09; as shown in Figure 6). Detailed characteristics of these meta-analyses and their assessment tools can be found in Supplementary Table S4 and Figure 4c, respectively.

CBT (*k* = 7), the most frequently examined intervention, was found to have little to no effect in reducing negative symptoms (*Median g* = −0.17; Range *g* = −0.46 to −0.09). For the remaining interventions, exercise therapy (*Median g* = −0.56; Range *g* = −0.56 to −0.55) and music therapy (*Median g* = −0.53; Range *g* = −0.56 to −0.50) had the largest effect sizes (as shown in Figure 6).

#### 3.2.4. Subgroup Analyses Within Each Symptom Dimension

Across the included meta-analyses, several subgroup findings offered insight into factors that may influence intervention effectiveness (as shown in Table 1). For music therapy, shorter treatment durations (<3 months versus ≥3 months) were generally associated with greater improvements in both total symptoms (*g* = −0.57; 95% CI = [−0.93, −0.20] versus *g* = −0.42; 95% CI = [−0.79, −0.05]) and positive symptoms (*g* = −0.53; 95% CI = [−0.96, −0.09] versus *g* = −0.15; 95% CI = [−0.46, 0.15]), although the difference was less pronounced for negative symptoms (*g* = −0.55; 95% CI = [−0.84, −0.26] versus *g* = −0.56; 95% CI = [−0.76, −0.36]). For horticultural therapy, interventions conducted in non-hospital environments (*g* = −2.62; 95% CI = [−3.87, −1.38]) showed larger reductions in total symptoms compared to those delivered in hospital settings (*g* = −0.90; 95% CI = [−1.21, −0.59]). Within CBT-focused analyses, positive symptom improvements tended to be larger among individuals with acute (*g* = −0.48; 95% CI = [−0.82, −0.13]) rather than chronic (*g* = −0.30; 95% CI = [−0.53, −0.07]) schizophrenia, and adapted CBT appeared more effective when delivered individually (*g* = −0.21; 95% CI = [−0.37, −0.05]) rather than in group (*g* = 0.17, 95% CI = [−0.09, 0.44]) formats for negative symptoms. Variability was also observed across patient populations, with inpatient groups (*g* = −0.20; 95% CI = [−0.57, 0.18]) showing somewhat greater improvement in negative symptoms than outpatient (*g* = −0.12; 95% CI = [−0.31, 0.08]) or mixed (*g* = −0.05; 95% CI = [−0.36, 0.25]) samples.

Taken together, these subgroup patterns suggest that intervention effectiveness is shaped not only by treatment modality, but also by contextual factors such as duration, setting, illness stage, delivery format, and patient population. Although the available subgroup data remain limited, these findings highlight the importance of tailoring psychological interventions to individual clinical profiles and service environments.

## 4. Discussion

### 4.1. General Discussion

Cognitive and behavioral interventions for schizophrenia have seen a rise in popularity both as an adjunctive treatment to antipsychotic medication and as a standalone treatment [28,29]. However, the existing literature on such interventions as a treatment for schizophrenia is largely restricted in terms of the type of intervention used, the symptom dimension of schizophrenia, the control condition(s) the treatment is being compared to, and the type of effect sizes used. This brings us to the purpose of this systematic umbrella review of meta-analyses which aims to bridge this gap by providing a comprehensive summary of the effectiveness of the different types of cognitive and behavioral interventions as a treatment for schizophrenia. Across the three symptom dimensions evaluated, median effect sizes of the different interventions were *g* = −0.38 (Range *g* = −1.56 to −0.08) for total symptom reduction, *g* = −0.30 (Range *g* = −0.84 to 0.00) for positive symptom reduction, and *g* = −0.39 (Range *g* = −0.66 to −0.09) for negative symptom reduction. These findings further solidify the current evidence supporting cognitive and behavioral interventions as effective treatments for schizophrenia. However, this effectiveness depends on the specific type of intervention used.

CBT was found to be the most commonly studied intervention used across all three dimensions of symptom-related outcomes, with *k* = 6 for total symptoms, *k* = 7 for positive symptoms, and *k* = 7 for negative symptoms. Regarding effectiveness, CBT was found to have a small to medium effect on the reduction in total symptoms (*Median g* = −0.31; Range *g* = −0.44 to −0.08) and positive symptoms (*Median g* = −0.31; Range *g* = −0.48 to −0.06), but little to no effect for negative symptoms (*Median g* = −0.17; Range *g* = −0.46 to −0.09). Across subgroup analyses, CBT demonstrated small variations in effectiveness across several contextual factors [77,78,79,80,81]. For positive symptoms, individuals with acute schizophrenia showed greater improvement than those with chronic illness [73]. For negative symptoms, individually delivered CBT appeared more effective than group-based formats [82]. Modest differences were also observed across patient populations, with inpatient samples showing slightly greater improvement in negative symptoms than outpatient or mixed samples [82]. These patterns suggest that although CBT is broadly effective, certain contextual factors may modestly influence its impact. When we look at how CBT works as a treatment for schizophrenia, its central notion revolves around the idea of engaging patients through a therapeutic alliance by helping them understand and re-evaluate their thoughts and feelings—about the symptoms they are experiencing—in an effort to help them feel more in control and adopt better coping skills [31,83]. In September 2020, CBT was further included as a psychosocial intervention treatment recommendation under “The Practice Guideline for Treatment of Patients diagnosed with Schizophrenia” by the American Psychiatric Association (APA) [84]. This addition thereby recognizes CBT as one of the primary treatment recommendations after antipsychotics. Concerning the comparatively limited effectiveness of CBT for negative symptoms, we refer to the discussion by Velthorst et al. [82], who highlighted the heterogeneous determinants of negative symptoms, including both expressive deficits and motivational or appraisal-based pathways [85]. They noted that trials selectively recruiting individuals with prominent negative symptoms may overrepresent “primary” negative symptoms—originating from expressive deficits and typically more resistant to CBT—relative to “secondary” negative symptoms that arise from dysfunctional expectancies and are potentially more amenable to cognitive–behavioral intervention [86,87].

Aside from CBT, we also looked at other individual cognitive and behavioral interventions that may prove to have beneficial effects for the treatment of schizophrenia. Within each symptom dimension of schizophrenia, there were a few interventions with considerably large effect sizes. For total symptoms, horticultural therapy (*g* = −1.56; 95% CI = [−2.08, −1.04]) [38] was found to have a large effect on total symptom reduction. According to the American Horticultural Therapy Association [88], horticultural therapy is defined as the active participation in horticultural activities that is facilitated by a trained horticultural therapist for the purpose of achieving specific treatment goals. The beneficial therapeutic effect of horticultural therapy stems from an individual’s interactions with plants, which aside from reducing psychopathological symptoms [89], also provide emotional benefits [90,91], enhance physical functioning [90,92], and improve quality of life [93,94]. It should be noted that the large effect size for horticultural therapy is derived from a single meta-analysis and should therefore be interpreted with caution.

For negative symptoms, exercise therapy (*Median g* = −0.56; Range *g* = −0.56 to −0.55) [64,77] and music therapy (*Median g* = −0.53; Range *g* = −0.56 to −0.50) [39,78] had a medium to large effect on negative symptom reduction. For exercise therapy, it involves physical activity that spans across a range of modalities such as but not limited to aerobic training and resistance training [64]. Engaging in such physical activities promotes overall cardiorespiratory fitness and quality of life while alleviating the issue of sedentary lifestyles faced by majority of those living with schizophrenia [95]. Whereas music therapy, when used as a supplement to standard care, acts as a non-verbal therapeutic medium that helps patients develop communication and expression [78]. This development further extends towards improved patient self-evaluation and quality of life [96,97]. The promise shown by interventions such as horticultural, exercise, and music therapies suggest the potential of more integrative and holistic approaches to therapeutic interventions for schizophrenia, not solely centered around cognitive restructuring but also involving lifestyle and environmental modifications [78,93]. While exercise therapy and music therapy showed relatively strong effects, these findings are currently supported by only two meta-analyses each, which warrants caution when interpreting the results.

For positive symptoms, family psychoeducation with patient behavioral and skills training had a medium to large effect on positive symptom reduction (*g* = −0.61; 95% CI = [−0.94, −0.28]) [58]. This form of intervention involves psychoeducation for both the patients and their relatives, combined alongside behavioral and skills training for patients alone. Family psychoeducation aims to establish an encouraging and collaborative relationship between all family members—including the individual with schizophrenia—to harness support in coping with common adversities [98,99,100], while behavioral and skills training aims to improve patient psychosocial and daily functioning [101,102,103]. The efficacy of family psychoeducation further underscores the important role of caregivers and the community as active participants in the treatment process of managing the symptoms of schizophrenia [104,105]. Although family psychoeducation combined with patient behavioral and skills training demonstrated a relatively large effect, this conclusion is drawn from a single meta-analysis and should therefore also be interpreted with due caution.

### 4.2. Clinical Implications and Future Directions

The findings of this umbrella review carry several implications for clinical practice. Although cognitive and behavioral interventions have been examined both as standalone treatments and as adjunctive components to pharmacotherapy, their clinical application typically favors an adjunctive role within comprehensive management plans for schizophrenia. Across the symptom domains synthesized in this review, these interventions demonstrated small to moderate improvements, indicating that they offer meaningful yet modest benefits when integrated into overall patient care. Furthermore, the differences across types of psychological interventions (e.g., cognitive remediation, social skills training), how they are delivered (e.g., individual vs. group sessions), and the specific symptoms they aim to address (e.g., positive symptoms, negative symptoms, or both) underscore the importance of tailoring treatments to each patient’s clinical profile and the resources available within different service settings. By consolidating the meta-analytic evidence, this umbrella review provides clinicians and service planners with clearer guidance on where these interventions show the strongest effects, informing more nuanced and evidence-based treatment decisions.

Despite the growing evidence supporting cognitive and behavioral interventions in schizophrenia, several important gaps remain. First, the variability observed across intervention types and symptom domains underscores the need for further research to identify which approaches are most effective for specific patient profiles. Second, little is known about factors that may influence treatment outcomes, such as baseline symptom severity, social functioning, or comorbidities, highlighting the importance of exploring moderators and mediators to guide more personalized care. Third, while most studies have evaluated traditional delivery formats, research on alternative modalities, including digital or hybrid approaches, remains limited and warrants investigation [106]. Addressing these gaps could inform the development of more targeted, evidence-based interventions and optimize functional outcomes for individuals living with schizophrenia.

### 4.3. Limitations

This umbrella review has several limitations worth acknowledging. First, as we specified certain types of control groups to increase comparability between meta-analyses, meta-analyses that directly compared the effectiveness of two or more cognitive and behavioral interventions against each other could not be included. Consequently, we were unable to draw direct conclusions about which intervention was potentially more effective than another. Future research could explicitly examine meta-analyses comparing interventions, which would further complement the findings of this review. Second, we were unable to analyze or report certain demographic information (e.g., mean age, gender distribution) due to inconsistent or missing data across the included meta-analyses, limiting our ability to explore whether participant characteristics influenced outcomes. Third, the high heterogeneity of the included meta-analyses restricted our ability to conduct subgroup analyses across studies, with analyses largely confined to those reported within each meta-analysis. Fourth, the quality and methodology of the included meta-analyses varied, and some relied on primary studies with small sample sizes or inconsistent outcome measures, which could affect the robustness of the synthesized findings. All included meta-analyses met the minimum methodological quality threshold based on the JBI critical appraisal checklist, indicating an overall adequate level of quality. However, variability in reporting standards, search rigor, and approaches to handling heterogeneity may influence the strength and interpretability of some findings. Fifth, data on moderators and mediators of treatment effects were limited, preventing a detailed understanding of factors influencing intervention efficacy. Finally, as with all umbrella reviews, our findings are dependent on the published literature and may be subject to publication bias, potentially overestimating intervention effects. These limitations should be considered when interpreting the findings and in guiding future primary research in this area.

## 5. Conclusions

This umbrella review demonstrates that cognitive and behavioral interventions are effective treatments for schizophrenia, with CBT showing the most robust evidence for reducing total and positive symptoms. Other approaches, including family psychoeducation with behavioral and skills training, exercise therapy, horticultural therapy, and music therapy, also offer meaningful benefits. The variability in intervention types and effects highlights the importance of tailoring treatments to individual patient profiles and service contexts. Overall, this review provides a comprehensive synthesis of current evidence to support informed, evidence-based clinical decision-making.

## Figures and Tables

**Table 1:** Characteristics of the Meta-analyses with Subgroup Analyses.

Author,	Type of Intervention *	Control Condition	Subgroup Type	Group	Number of Studies	Sample Size	Hedges’ *g* (95% CI)
Publication Year							
***Total symptoms***							
Jia et al. (2020) [39]	MT	TAU, WLC, Medication	Duration of MT	<3 months	6	-	−0.57 [−0.93, −0.20]
				≥3 months	7	-	−0.42 [−0.79, −0.05]
Lu et al. (2021) [38]	HT	TAU	Type of environment	Hospital environment	10	983	−0.90 [−1.21, −0.59]
				Non-hospital environment	7	470	−2.62 [−3.87, −1.38]
***Positive symptoms***							
Jia et al. (2020) [39]	MT	TAU, WLC, Medication	Duration of MT	<3 months	4	-	−0.53 [−0.96, −0.09]
				≥3 months	8	-	−0.15 [−0.46, 0.15]
Zimmerman et al. (2005) [73]	CBT	TAU	Type of condition	Chronic	6	569	−0.30 [−0.53, −0.07]
				Acute	2	133	−0.48 [−0.82, −0.13]
***Negative Symptoms***							
Jia et al. (2020) [39]	MT	TAU, WLC, Medication	Duration of MT	<3 months	6	-	−0.55 [−0.84, −0.26]
				≥3 months	9	-	−0.56 [−0.76, −0.36]
Velthorst et al. (2015) [82]	Adapted CBT	TAU	Treatment setting	Group	8	602	0.17 [−0.09, 0.44]
				Individual	20	1486	−0.21 [−0.37, −0.05]
			Study population	Outpatient	17	1227	−0.12 [−0.31, 0.08]
				In and outpatient	7	585	−0.05 [−0.36, 0.25]
				Inpatient	4	276	−0.20 [−0.57, 0.18]

* MT: Music therapy; TAU: Treatment as usual; WLC: Wait-list control; HT: Horticultural therapy; CBT: Cognitive behavioral therapy.

## Data Availability

No new data were created or generated in this study. All data analyzed in this umbrella review were derived from previously published meta-analyses. Supporting materials, including extracted data tables, are provided in the Supplementary Materials and are also publicly available on the Open Science Framework (OSF; Center for Open Science, Charlottesville, VA, USA; https://osf.io/r2ja7/, accessed on 13 December 2025).

## Supplementary Materials

The following supporting information can be downloaded at: https://www.mdpi.com/article/10.3390/jcm15010187/s1, Table S1: Inter-Rater Agreement Rates for Each Stage of the Umbrella Review; Table S2: Characteristics of the Meta-analyses Measuring the Reduction of Total Symptoms; Table S3: Characteristics of the Meta-analyses Measuring the Reduction of Positive Symptoms; Table S4: Characteristics of the Meta-analyses Measuring the Reduction of Negative Symptoms; Data extraction file; Full-text screening file; Quality assessment (for included meta-analyses); Quality assessment (for excluded meta-analyses); Title and abstract screening file; PRISMA Checklist [107]; Regions and countries represented in the included meta-analyses; Excluded meta-analyses (with reasons).
