# Supplementary material for: Effectiveness of Cognitive and Behavioral Interventions in the Treatment of Schizophrenia: An Umbrella Review of Meta-Analyses
Source: J Clin Med. 2025 Dec 26;15(1):187. doi: 10.3390/jcm15010187 (PMC12786704; doi:10.3390/jcm15010187)
Supplement: Supplementary file 1 [file jcm-15-00187-s001.zip › Excluded meta-analyses (with reasons).pdf]

Author, Year of publication	Contacted author(s)?	Received a response?	Reasons for exclusion
Cui et al. (2024)	Yes	No	The meta-analysis reported effect sizes as overall mean differences which could not be converted to our effect size of interest (Hedges' *g*). Additionally, there was also insufficient data available for us to perform a manual effect size calculation.
Guo et al. (2024)	Yes	No	The meta-analysis reported effect sizes as overall mean differences which could not be converted to our effect size of interest (Hedges' *g*). Additionally, there was also insufficient data available for us to perform a manual effect size calculation.
Mueller et al. (2013)	Yes	No	The meta-analysis reported an overall weighted effect size which could not be converted to our effect size of interest (Hedges' *g*). Additionally, there was also insufficient data available for us to perform a manual effect size calculation.
Roder et al. (2006)	Yes	No	The meta-analysis reported an overall weighted effect size which could not be converted to our effect size of interest (Hedges' *g*). Additionally, there was also insufficient data available for us to perform a manual effect size calculation.
Su et al. (2024)	Yes	No	The meta-analysis reported effect sizes as overall mean differences which could not be converted to our effect size of interest (Hedges' *g*). Additionally, there was also insufficient data available for us to perform a manual effect size calculation.
Tseng et al. (2016)	Yes	Yes	The meta-analysis reported a Hedges' *g* that was implausibly large, which warranted further investigation of the empirical studies included. Upon closer inspection, we noted that the effect sizes obtained from some of the empirical studies were converted through other means (e.g., calculating effect sizes from *t*-test scores).<br><br>Upon clarification by the author(s), we were told that the software Comprehensive Meta-Analysis (CMA) version 2.0 was used for the calculation/conversion of the effect sizes. As the software used was an older version, we attempted to contact the CMA software team for clarification about how the effect

			sizes were calculated—but did not receive the required information.
Wykes et al. (2008)	Yes	Yes	The meta-analysis reported an overall weighted effect size which could not be converted to our effect size of interest (Hedges' *g*). Additionally, there was also insufficient data available for us to perform a manual effect size calculation.<br><br>Upon clarification by the author(s), we were told that they no longer had the information we required.

References

Cui, W., Liu, Z., Liang, C., & Zhao, Z. (2024). Comparative efficacy of different types of exercise modalities on psychiatric symptomatology in patients with schizophrenia: A systematic review with network meta-analysis. *Scientific Reports*, *14*(1), 7019. https://doi.org/10.1038/s41598-024-57081-3

Guo, J., Liu, K., Liao, Y., Qin, Y., & Yue, W. (2024). Efficacy and feasibility of aerobic exercise interventions as an adjunctive treatment for patients with schizophrenia: A meta-Analysis. *Schizophrenia*, *10*(1), 2. https://doi.org/10.1038/s41537-023-00426-0

Müller, D., Schmidt, S. J., & Roder, V. (2013). *Integrated psychological therapy: Effectiveness in schizophrenia inpatient settings related to patients' age*. https://doi.org/10.7892/BORIS.17372

Roder, V. (2006). Integrated Psychological Therapy (IPT) for Schizophrenia: Is It Effective? *Schizophrenia Bulletin*, *32*(Supplement 1, S81–S93. https://doi.org/10.1093/schbul/sbl021

Su, Y., Pan, X., Li, H., & Zhang, G. (2024). Effects of mind-body therapies on schizophrenia: A systematic review and network meta-analysis. *Schizophrenia Research*, *264*, 236–247. https://doi.org/10.1016/j.schres.2023.12.030

Tseng, P.-T., Chen, Y.-W., Lin, P.-Y., Tu, K.-Y., Wang, H.-Y., Cheng, Y.-S., Chang, Y.-C., Chang, C.-H., Chung, W., & Wu, C.-K. (2016). Significant treatment effect of adjunct music therapy to standard treatment on the positive, negative, and mood symptoms of schizophrenic patients: A meta-analysis. *BMC Psychiatry*, *16*(1), 16. https://doi.org/10.1186/s12888-016-0718-8

Wykes, T., Steel, C., Everitt, B., & Tarrier, N. (2007). Cognitive Behavior Therapy for Schizophrenia: Effect Sizes, Clinical Models, and Methodological Rigor. *Schizophrenia Bulletin*, *34*(3), 523–537. https://doi.org/10.1093/schbul/sbm114
