# Supplementary material for: Effectiveness of Cognitive and Behavioral Interventions in the Treatment of Schizophrenia: An Umbrella Review of Meta-Analyses
Source: J Clin Med. 2025 Dec 26;15(1):187. doi: 10.3390/jcm15010187 (PMC12786704; doi:10.3390/jcm15010187)
Supplement: Supplementary file 1 [file jcm-15-00187-s001.zip › Regions and Countries Represented in the Included Meta.pdf]

Regions and Countries Represented in the Included Meta-Analyses

Asia-Pacific: Australia, China, Hong Kong, India, Indonesia, Japan, Korea, Malaysia, Pakistan, South Korea, Sri Lanka, Taiwan, Thailand

Europe: Austria, Belgium, Denmark, England, Finland, France, Germany, Greece, Ireland, Italy, Netherlands, Norway, Poland, Portugal, Serbia, Spain, Switzerland, Turkey, UK

Middle East: Egypt, Iran, Saudi Arabia

North America: Canada, USA

South America: Brazil, Mexico
