# Supplementary material for: Effectiveness of Cognitive and Behavioral Interventions in the Treatment of Schizophrenia: An Umbrella Review of Meta-Analyses
Source: J Clin Med. 2025 Dec 26;15(1):187. doi: 10.3390/jcm15010187 (PMC12786704; doi:10.3390/jcm15010187)
Supplement: Supplementary file 1 [file jcm-15-00187-s001.zip › Table S1. Inter-Rater Agreement Rates for Each Stage of the Umbrella Review.pdf]

**Table S1**

*Inter-Rater Agreement Rates for Each Stage of the Umbrella Review*

| Stage | Agreement (%) |
|---|---|
| **Screening (based on abstracts)** | |
| English | 99 |
| Mentioned schizophrenia or related disorders | 87 |
| Mentioned psychotherapy | 88 |
| Meta-analysis | 90 |
| **Screening (based on full texts)** | |
| English | 98 |
| Meta-analysis | 96 |
| At least five studies | 77 |
| Population with schizophrenia or related disorders | 85 |
| Effectiveness of cognitive and behavioral intervention | 79 |
| Reported symptom-related outcomes | 74 |
| Suitable control condition | 78 |
| *Quality assessment* | |
| Clear review question | 100 |
| Appropriate inclusion criteria[a] | 100 |
| Appropriate search strategy | 97 |
| Adequate use of sources[b] | 91 |
| Appropriate appraisal criteria | 94 |
| Appraisal by two or more reviewers | 84 |
| Methods to minimize error in data extraction[c] | 88 |
| Appropriate method to combine studies | 100 |
| Assessed publication bias | 84 |
| Appropriate recommendations | 100 |
| Appropriate new research directives | 88 |
| **Data extraction** | |
| Sample demographics | 100 |
| Year(s) searched for studies | 89 |
| Regions covered | 89 |
| Total number of studies | 86 |
| Total sample size | 82 |
| Type of cognitive and behavioral intervention | 96 |
| Effect size | 82 |
| $I^2$ values | 100 |
| How overall effect size was estimated | 86 |
| Assessment tools used | 89 |
| Number of studies for subgroup analysis (if any) | 86 |
| Subgroup analysis in detail (if any) | 86 |
| Limitations | 96 |

[a] Appropriate inclusion consists of populations that are schizophrenic, capture interventions that are psychological, and select for suitable control conditions
[b] Sources should capture both published and unpublished literature
[c] These include methods such as but not limited to, having two independent coders, performing the coding twice, and performing checks
