# Supplementary material for: Effectiveness of Cognitive and Behavioral Interventions in the Treatment of Schizophrenia: An Umbrella Review of Meta-Analyses
Source: J Clin Med. 2025 Dec 26;15(1):187. doi: 10.3390/jcm15010187 (PMC12786704; doi:10.3390/jcm15010187)
Supplement: Supplementary file 1 [file jcm-15-00187-s001.zip › Table S2-S4. Characteristics of the included meta-analyses.pdf]

Table S2

Characteristics of the Meta-analyses Measuring the Reduction of Total Symptoms

Author, Publication year	Sample diagnosis criteria	Sample type (Inpatient/ Outpatient)	Country, Region	Type of intervention	Control condition	Number of studies	Sample size	Hedges' *g* (95% CI)	I^2	Assessment tools
Bighelli et al. (2018)	Schizophrenia and related disorders (NA)	Mixed	16 countries (Asia-Pacific, Europe, Middle East, North America)	CBT	TAU	15	1208	-0.36 [-0.54, -0.17]	-	PANSS, BPRS
Burlingame et al. (2020)	Schizophrenia, psychosis, and related disorders (DSM; ICD)	Not stated	4 countries (Europe, North America)	CBT	TAU, WLC, AttCG	5	462	-0.08 [-0.39, 0.23]	0%	PANSS, BPRS
			9 countries (Asia-Pacific, Europe, North America)	CR	TAU, WLC, AttCG, Medication	17	852	-0.31 [-0.51, -0.11]	1%	
			4 countries (Asia-Pacific, Europe, North America)	MFG	TAU, WLC	5	420	-0.39 [-0.71, -0.07]	41%	
			5 countries (Asia-Pacific, Europe, North America, South America)	SS	TAU, WLC, Medication	11	615	-0.53 [-0.77, -0.29]	75%	
Degnan et al. (2018)	Schizophrenia or schizoaffective disorder (DSM; ICD)	Mixed	13 countries (Asia-Pacific, Europe, Middle East, North America,	CBT, CaCBT, MCT, SS, FI	Standard care and medication	9	1581	-0.24 [-0.41, -0.06]	43%	PANSS, BPRS, SAPS, SANS

			South America)							
Gray et al. (2016)	Schizophrenia and related disorders (DSM)	Mixed	8 countries (Asia-Pacific, Europe, North America)	AT	TAU	6	707	-0.56 [-1.03, -0.09]	86%	PANSS, BPRS
Guaiana et al. (2022)	Schizophrenia and related disorders (any)	Mixed	3 countries (Asia-Pacific, Europe, North America)	Group CBT	Standard care	12	1036	-0.44 [-0.65, -0.22]	5%	PANSS
Jauhar et al. (2014)	Schizophrenia, schizoaffective or non-affective functional psychosis (NA)	Not stated	-	CBT	TAU	21	1992	-0.33 [-0.45, -0.21]	-	PANSS, BPRS, SAPS, CPRS, KMS
Jia et al. (2020)	Schizophrenia and related disorders (any)	Inpatient	5 countries (Asia-Pacific, Europe)	MT	TAU, WLC, Medication	13	911	-0.48 [-0.74, -0.22]	72%	PANSS, BPRS
Li et al. (2023)	Schizophrenia and related disorders (NA)	Mixed	7 countries (Asia-Pacific, Europe)	AT	TAU	5	726	-0.69 [-1.33, -0.05]	92%	PANSS, BPRS
Lu et al. (2021)	Schizophrenia (any)	Mixed	China, Korea	HT	TAU	17	1453	-1.56 [-2.08, -1.04]	94%	PANSS, BPRS
Oliva et al. (2023)	Psychotic disorders (NA)	Not stated	11 countries (Asia-Pacific, Europe, Middle East, North America, South America)	ExT	Standard care	12	380	-0.28 [-0.55, 0.00]	60%	PANSS
Orfanos et al. (2015)	Schizophrenia and related disorders (DSM; ICD)	Mixed	-	Group-based: CBT, CR, MCT, SS, ArT, MT, PE, Other	TAU	9	651	-0.41 [-0.69, -0.13]	60.30%	PANSS, BPRS

Polese et al. (2019)	Treatment-resistant schizophrenia or treatment-resistant psychosis	Mixed	-	CBT, MCT, ST	TAU	5	389	-0.22 [-0.44, 0.00]	46%	PANSS
Qin et al. (2024)	Schizophrenia (DSM)	Mixed	6 countries (Asia-Pacific, Europe)	MBI	TAU, WLC, Medication, No treatment	9	906	-1.08 [-1.23, -0.93]	94.40%	PANSS, BPRS
Rodolico et al. (2022)	Schizophrenia and related disorders (DSM; ICD)	Mixed	-	FPEBST	TAU	9	1501	-0.61 [-0.93, -0.29]	-	PANSS
Rosenbaum et al. (2014)	Schizophrenia or schizoaffective disorder (any)	Mixed	-	ExT	TAU, WLC, No treatment	8	373	-1.00 [-1.64, -0.37]	87.90%	PANSS, SAPS, SANS
Salahuddin et al. (2024)	Schizophrenia and related disorders (NA)	Mixed	3 countries (Europe, North America)	CBT	TAU	10	1586	-0.22 [-0.35, -0.09]	-	PANSS, BPRS, SCS
Swanson (2024)	Schizophrenia and related disorders (NA)	Mixed	-	CBT	TAU, WLC	18	-	-0.29 [-0.42, -0.16]	36%	-
			-	CR	TAU, WLC	7	-	-0.25 [-0.50, 0.00]	42%	
			-	ArT	TAU, WLC	6	-	-0.41 [-0.82, 0.01]	82%	
			-	MBI	TAU, WLC	8	-	-0.43 [-0.71, -0.15]	65%	
Wykes et al. (2011)	Schizophrenia (NA)	Mixed	-	CR	Standard care and medication	20	1114	-0.18 [-0.32, -0.03]	-	PANSS, BPRS, SAPS, CPRS, PSYRATS

Note. NA: Not available; CBT: Cognitive behavioral therapy; TAU: Treatment as usual; PANSS: Positive and Negative Syndrome Scale; BPRS: Brief Psychiatric Rating Scale; SCS: Strauss-Carpenter Scale; WLC: Wait-list control; AttCG: Attention control group; DSM: Diagnostic and Statistical Manual (any edition); ICD: International Classification of Diseases (any edition); CR: Cognitive remediation; MFG: Multifamily group therapy; SS: Social skills training; MCT: Metacognitive training; FI: Family

intervention therapies; CaCBT: Culturally-adapted cognitive behavioral therapy; SAPS: Scale for the assessment of positive symptoms; SANS: Scale for the assessment of negative symptoms; AT: Adherence therapy; ArT: Art therapy; HT: Horticultural therapy; PE: Psychoeducation; ST: Supportive therapy; ExT: Exercise therapy; CPRS: Comprehensive psychopathology rating scale; KMS: Krawiecka (Manchester) scale; PSYRATS: Psychotic symptom rating scale; MT: Music therapy; FPEBST: Family psychoeducation with patient behavioral and skills training, MBI: Mindfulness-based interventions

Table S3

Characteristics of the Meta-analyses Measuring the Reduction of Positive Symptoms

**Author, Publication year**	**Sample diagnosis criteria**	**Sample type (Inpatient/ Outpatient)**	**Country, Region**	**Type of intervention**	**Control condition**	**Number of studies**	**Sample size**	**Hedges' *g* (95% CI)**	**I^2**	**Assessment tools**
Barnicot et al. (2020)	Schizophrenia and related disorders (ICD)	Inpatient	-	CBT, MCT, MotT, PE, ACT, EMDR	TAU	9	483	-0.32 [-0.61, -0.04]	52.80%	-
Bighelli et al. (2018)	Schizophrenia and related disorders (NA)	Mixed	16 countries (Asia-Pacific, Europe, Middle East, North America)	CBT	TAU	18	1817	-0.30 [-0.45, -0.14]	-	PANSS, BPRS
Broderick et al. (2015)	Schizophrenia and related disorders (NA)	Mixed	4 countries (Asia-Pacific, North America)	YogT	Standard care	5	243	-0.30 [-0.56, -0.04]	0%	PANSS

Degnan et al. (2018)	Schizophrenia or schizoaffective disorder (DSM; ICD)	Mixed	13 countries (Asia-Pacific, Europe, Middle East, North America, South America)	CBT, CaCBT, MCT, SS, FI	Standard care and medication	7	449	-0.84 [-1.29, -0.38]	79%	PANSS, BPRS, SAPS
Eichner & Berna (2016)	Schizophrenia and related disorders (DSM; ICD)	Not stated	-	MCT	TAU, WLC	7	254	-0.38 [-0.67, -0.08]	-	PANSS
Guaiana et al. (2022)	Schizophrenia and related disorders (any)	Mixed	United Kingdom, United States of America	Group CBT	Standard care	8	539	-0.06 [-0.23, 0.10]	0%	PANSS
Hodann-Caudevilla et al. (2020)	Schizophrenia and related disorders (DSM; ICD)	Not stated	4 countries (Asia-Pacific, North America)	PBCT, MBPE, MBCT, MIRRORS, MBI	TAU, WLC	5	558	-0.32 [-0.60, -0.04]	99%	PANSS, BPRS, PSYRATS
Jauhar et al. (2014)	Schizophrenia, schizoaffective or non-affective functional psychosis (NA)	Not stated	-	CBT	TAU	19	1535	-0.31 [-0.45, -0.17]	-	PANSS, BPRS, SAPS, CPRS, KMS
Jia et al. (2020)	Schizophrenia and related disorders (any)	Mixed	5 countries (Asia-Pacific, Europe)	MT	TAU, WLC, Medication	12	791	-0.27 [-0.54, 0.00]	70%	PANSS, BPRS
Naeem et al. (2016)	Schizophrenia and related disorders (NA)	Not stated	-	CBT	TAU	5	1019	-0.48 [-0.73, -0.22]	40.37%	PANSS, CPRS

Oliva et al. (2023)	Psychotic disorders (NA)	Not stated	11 countries (Asia-Pacific, Europe, Middle East, North America, South America)	ExT	Standard care	16	614	-0.29 [-0.47, -0.12]	49%	PANSS
Orfanos et al. (2015)	Schizophrenia and related disorders (DSM; ICD)	Mixed	-	Group-based: CBT, CR, MCT, SS, ArT, MT, PE, Other	TAU	11	730	-0.06 [-0.25, 0.13]	29.80%	PANSS, BPRS, PECC
Philipp et al. (2019)	Schizophrenia (DSM; ICD)	Mixed	10 countries (Asia-Pacific, Europe)	MCI	Standard treatment	11	506	-0.27 [-0.59, 0.05]	60.70%	PANSS, PSYRATS
Rodolico et al. (2022)	Schizophrenia and related disorders (DSM; ICD)	Mixed	-	FPEBST	TAU	9	774	-0.61 [-0.94, -0.28]	-	PANSS
Salahuddin et al. (2024)	Schizophrenia and related disorders (NA)	Mixed	3 countries (Europe, North America)	CBT	TAU	15	1838	-0.31 [-0.43, -0.19]	-	PANSS, BPRS
Swanson (2024)	Schizophrenia and related disorders (NA)	Mixed	-	CBT	TAU, WLC	18	-	-0.23 [-0.37, -0.09]	47%	-
			-	CR	TAU, WLC	9	-	-0.25 [-0.50, 0.00]	60%	
			-	ArT	TAU, WLC	7	-	-0.20 [-0.51, 0.10]	74%	
			-	MCT	TAU, WLC	5	-	-0.58 [-0.87, -0.28]	0%	
			-	MBI	TAU, WLC	6	-	-0.17 [-0.42, 0.08]	-	

			-	SS	TAU, WLC	6	-	0.00 [-0.20, 0.21]	-	
Wei et al. (2020)	Schizophrenia (NA)	Mixed	-	MBET	TAU	7	401	-0.29 [-0.55, -0.03]	36%	PANSS, SAPS
Yin et al. (2024)	Schizophrenia (NA)	Mixed	7 countries (Asia-Pacific, Europe, North America)	YogT	TAU, WLC	12	639	-0.37 [-0.62, -0.13]	56%	PANSS, SAPS
Zimmerman et al. (2005)	Schizophrenia and related disorders (DSM)	Not stated	-	CBT	TAU	8	702	-0.32 [-0.50, -0.15]	-	PANSS, BPRS, CPRS, SAPS, PSYRATS

Note. NA: Not available; CBT: Cognitive behavioral therapy; TAU: Treatment as usual; PANSS: Positive and Negative Syndrome Scale; BPRS: Brief Psychiatric Rating Scale; KMS: Krawiecka (Manchester) scale; WLC: Wait-list control; AttCG: Attention control group; DSM: Diagnostic and Statistical Manual (any edition); ICD: International Classification of Diseases (any edition); SS: Social skills training; MCT: Metacognitive training; FI: Family intervention therapies; CaCBT: Culturally-adapted cognitive behavioral therapy; SAPS: Scale for the assessment of positive symptoms; PE: Psychoeducation; ExT: Exercise therapy; CPRS: Comprehensive psychopathology rating scale; PSYRATS: Psychotic symptom rating scale; MotT: Motivational therapy; ACT: Acceptance and commitment therapy; EMDR: Eye movement desensitization and reprocessing; PBCT: Person-based cognitive therapy; MBPE: Mindfulness-based psychoeducation; MBCT: Mindfulness-based cognitive therapy; MIRRORS: Mindfulness intervention for rehabilitation and recovery in schizophrenia; MBI: Mindfulness-based interventions; IPT: Integrated psychological therapy; PECC: Psychosis evaluation tool for common use by caregivers; MCI: Metacognitive interventions; YogT: Yoga therapy; MBET: Mind-body exercise therapy; ArT: Art therapy; MT: Music therapy; FPEBST: Family psychoeducation with patient behavioral and skills training

Table S4

Characteristics of the Meta-analyses Measuring the Reduction of Negative Symptoms

**Author, Publication year**	**Sample diagnosis criteria**	**Sample type (Inpatient/ Outpatient)**	**Country, Region**	**Type of intervention**	**Control condition**	**Number of studies**	**Sample size**	**Hedges' *g* (95% CI)**	**I^2**	**Assessment tools**
Bighelli et al. (2018)	Schizophrenia and related disorders (NA)	Mixed	16 countries (Asia-Pacific, Europe, Middle East, North America)	CBT	TAU	11	916	-0.15 [-0.29, -0.02]	-	PANSS, BPRS
Broderick et al. (2015)	Schizophrenia and related disorders (NA)	Mixed	4 countries (Asia-Pacific, North America)	YogT	Standard care	5	243	-0.36 [-0.85, 0.13]	74.73%	PANSS
Cella et al. (2017)	Schizophrenia or schizoaffective disorder (DSM; ICD)	Not stated	-	CR	TAU	16	872	-0.36 [-0.52, -0.20]	-	PANSS, BPRS, SANS
Degnan et al. (2018)	Schizophrenia or schizoaffective disorder (DSM; ICD)	Mixed	13 countries (Asia-Pacific, Europe, Middle East, North America, South America)	CBT, CaCBT, MCT, SS, FI	Standard care and medication	7	449	-0.66 [-0.89, -0.44]	23%	PANSS, BPRS, SANS
Fusar-Poli et al. (2015)	Schizophrenia or schizoaffective disorder (DSM; ICD)	Not stated	-	CBT, ExT, YogT, ArT, MT, SS, FI, CT, SC	TAU, Placebo-AttCG	27	980	-0.40 [-0.56, -0.23]	57.60%	PANSS, BPRS, SANS

Geretsegger et al. (2017)	Schizophrenia and related disorders (any)	Mixed	-	MT	Standard care	5	319	-0.50 [-0.73, -0.27]	67.07%	SANS
Guaiana et al. (2022)	Schizophrenia and related disorders (any)	Mixed	4 countries (Asia-Pacific, Europe, North America)	Group CBT	Standard care	9	768	-0.20 [-0.46, 0.07]	65%	PANSS
Hodann-Caudevilla et al. (2020)	Schizophrenia and related disorders (DSM; ICD)	Not stated	6 countries (Asia-Pacific, Europe, North America)	PBCT, MBPE, MBCT, MIRRORS, MBI	TAU, WLC	5	558	-0.40 [-0.51, -0.29]	82%	PANSS, BPRS, SANS
Jauhar et al. (2014)	Schizophrenia, schizoaffective or non-affective functional psychosis (NA)	Not stated	-	CBT	TAU	20	1431	-0.17 [-0.33, -0.02]	-	PANSS, BPRS, CPRS, KMS
Jia et al. (2020)	Schizophrenia and related disorders (any)	Mixed	5 countries (Asia-Pacific, Europe)	MT	TAU, WLC, Medication	15	964	-0.56 [-0.72, -0.40]	30%	PANSS, BPRS
Oliva et al. (2023)	Psychotic disorders (NA)	Not stated	11 countries (Asia-Pacific, Europe, Middle East, North America, South America)	ExT	Standard care	16	614	-0.56 [-1.02, -0.10]	73%	PANSS
Orfanos et al. (2015)	Schizophrenia and related disorders (DSM; ICD)	Mixed	-	Group-based: CBT, CR, MCT, SS, ArT, MT, PE, Other	TAU	15	893	-0.37 [-0.60, -0.14]	59.80%	PANSS, SANS

Riehle et al. (2020)	Schizophrenia and related disorders (DSM; ICD)	Not stated	5 countries (Asia-Pacific, Europe, North America)	CBT	Standard treatment	6	358	-0.46 [-0.77, -0.15]	50%	PANSS, SANS, BNSS, NSA
Rodolico et al. (2022)	Schizophrenia and related disorders (DSM; ICD)	Mixed	-	FPEBST	TAU	9	774	-0.43 [-0.65, -0.20]	-	PANSS
Salahuddin et al. (2024)	Schizophrenia and related disorders (NA)	Mixed	3 countries (Europe, North America)	CBT	TAU	6	997	-0.14 [-0.29, -0.01]	-	PANSS, BPRS
Swanson (2024)	Schizophrenia and related disorders (NA)	Mixed	-	CBT	TAU, WLC	19	-	-0.24 [-0.38, -0.10]	41%	-
			-	CR	TAU, WLC	9	-	-0.27 [-0.44, -0.10]	23%	
			-	ArT	TAU, WLC	11	-	-0.48 [-0.77, -0.19]	79%	
			-	MBI	TAU, WLC	6	-	-0.24 [-0.53, 0.05]	59%	
			-	SS	TAU, WLC	7	-	-0.24 [-0.47, -0.01]	20%	
Velthorst et al. (2015)	Schizophrenia (NA)	Mixed	10 countries (Asia-Pacific, Europe, North America)	Adapted CBT	TAU	28	2088	-0.09 [-0.21, 0.03]	62.70%	PANSS, BPRS, SANS, BRIANS
Vogel et al. (2019)	Schizophrenia and related disorders (NA)	Mixed	7 countries (Asia-Pacific, Europe, North America)	ExT	TAU	16	1357	-0.55 [-0.85, -0.25]	80%	PANSS, SANS
Wei et al. (2020)	Schizophrenia (NA)	Mixed	-	MBET	TAU	8	697	-0.43 [-0.65, -0.20]	46%	PANSS, SANS

Yin et al. (2024)	Schizophrenia (NA)	Mixed	7 countries (Asia-Pacific, Europe, North America)	YogT	TAU, WLC	12	639	-0.51 [-0.69, -0.33]	56%	PANSS, SANS

Note. NA: Not available; CBT: Cognitive behavioral therapy; TAU: Treatment as usual; PANSS: Positive and Negative Syndrome Scale; BPRS: Brief Psychiatric Rating Scale; CR: Cognitive remediation; WLC: Wait-list control; AttCG: Attention control group; SANS: Scale for the assessment of negative symptoms; KMS: Krawiecka (Manchester) scale; DSM: Diagnostic and Statistical Manual (any edition); ICD: International Classification of Diseases (any edition); SS: Social skills training; MCT: Metacognitive training; FI: Family intervention therapies; CaCBT: Culturally-adapted cognitive behavioral therapy; PE: Psychoeducation; ExT: Exercise therapy; CPRS: Comprehensive psychopathology rating scale; PBCT: Person-based cognitive therapy; MBPE: Mindfulness-based psychoeducation; MBCT: Mindfulness-based cognitive therapy; MIRRORS: Mindfulness intervention for rehabilitation and recovery in schizophrenia; MBI: Mindfulness-based interventions; MBET: Mind-body exercise therapy; YogT: Yoga therapy; ArT: Art therapy; CT: Cognitive therapy; SC: Supportive counseling; MT: Music therapy; BNSS: Brief negative symptom scale; NSA: Negative symptom assessment; BRIANS: Brief assessment of negative symptoms scale; FPEBST: Family psychoeducation with patient behavioral and skills training.
